# Diversity and composition of soil microbial communities in the rhizospheres of late blight-resistant tomatoes after *Phytophthora infestans* inoculation

**DOI:** 10.3389/fpls.2025.1556928

**Published:** 2025-03-07

**Authors:** Xinyan Zhou, Liyuan Liao, Ken Chen, Yan Yin, Lulu Qiu, Xinni Li, Qingshan Li, Shangdong Yang

**Affiliations:** Guangxi Key Laboratory of Agro-environment and Agro-products Safety, National Demonstration Center for Experimental Plant Science Education, College of Agriculture, Guangxi University, Nanning, Guangxi, China

**Keywords:** tomato, *Solanum lycopersicum* L., late blight, soil microbial community structure, rhizosphere, antagonistic microorganisms

## Abstract

Late blight caused by the oomycete *Phytophthora infestans* poses a severe threat to global tomato (*Solanum lycopersicum* L.) production. While genetic resistance forms the cornerstone of disease control, the mechanisms underlying cultivar-specific resistance, particularly their interactions with rhizosphere microbiomes, remain poorly understood. To elucidate the mechanisms of tomato cultivar resistance to late blight and screen out antagonistic microorganisms against *P*. *infestans*, we investigated the microbial compositions in the rhizospheres of tomato cultivars with different late blight-resistance levels under both natural and *P. infestans*-inoculated conditions. Considerable differences in soil microbial diversity and composition of rhizospheres were found between late blight-resistant and -susceptible tomato cultivars. Under natural conditions, the resistant tomato cultivar exhibited higher bacterial diversity and lower fungal diversity than that of the susceptible cultivar. Additionally, after *P. infestans* inoculation, both the resistant and susceptible cultivars showed enrichment of microorganisms with potential antagonistic effects in the rhizospheres. Among them, bacterial genera, such as *Pseudomonas*, *Azospirillum*, and *Acidovorax*, and fungal genera, including *Phoma*, *Arthrobotrys*, *Pseudallescheria*, and *Pseudolabrys*, were enriched in the rhizospheres of the late blight-resistant tomato cultivar. In contrast, bacterial genera, including *Flavobacterium*, *Pseudolabrys*, and *Burkholderia-Caballeronia-Paraburkholderia*, and the *Trichoderma* fungal genus were enriched in the rhizospheres of the late blight-susceptible tomato cultivar. Simultaneously, the enrichment of pathogenic microorganisms, such as *Neocosmospora* and *Plectosphaerella*, was also detected in the rhizospheres of the susceptible tomato cultivar. Moreover, no enrichment of pathogenic microorganisms occurred in the late blight-resistant tomato cultivar after *P. infestans* inoculation. These findings suggest that these traits serve as effective defense mechanisms against pathogen invasion in resistant tomato cultivar. Overall, this study provides a comprehensive analysis of the rhizosphere microbial community structures in late blight-resistant and -susceptible tomato cultivars under natural conditions and their response following pathogen inoculation. Additionally, potential antagonistic microorganisms against late blight were also identified. The findings offer valuable insights for effective late blight management in tomatoes and contribute to the development of sustainable agricultural practices.

## Introduction

Tomato (*Solanum lycopersicum* L.) is a crop of considerable economic importance worldwide, and it continues to grow in terms of both cultivation areas and yields in China. However, late blight caused by the pathogenic *Phytophthora infestans* poses a serious threat to sustainable tomato production. This disease is highly destructive and causes large-scale crop losses, with severe economic consequences for agricultural production (Arafa et al., 2022). Currently, the main strategies for controlling late blight, both domestically and internationally, include physical control, chemical treatment, and the development of disease-resistant cultivars (Ivanov et al., 2021). However, traditional methods have several limitations. Physical control is cumbersome and inefficient; hence, implementation on a large scale is challenging. Although chemical control through pesticide application effectively suppresses diseases in the short term, prolonged use results in environmental pollution and potential risks to food safety (Liu et al., 2018). Furthermore, excessive pesticide use triggers pathogens to develop resistance, thereby enhancing their mutation rates and pathogenicity (Tang et al., 2021). Although breeding disease-resistant cultivars is an ideal long-term strategy, the process is time-consuming and often takes several years or even decades (Galeano Garcia et al., 2018). Therefore, low-cost, environmentally friendly, and sustainable solutions for controlling late blight are urgently required. Among these alternatives, biological control has gained notable attention because of its eco-friendly and sustainable nature. Antagonistic microorganisms and microbial inoculants are essential for controlling plant diseases in sustainable agriculture (Azcón-Aguilar and Barea, 1997). Therefore, exploring beneficial microbe-plant interactions and their application in agricultural production is critical because it may reduce the need for fertilizers and pesticides, which subsequently contributes to more sustainable agricultural practices (Qu et al., 2020).

The rhizosphere microbiome is often referred to as the “second genome” or “extended genotype” of the plant (Banerjee and van der Heijden, 2023). Rhizosphere microorganisms play a crucial role in promoting plant growth and development, particularly by facilitating the absorption of mineral nutrients, enhancing plant disease resistance, and improving tolerance to abiotic and biotic stressors (Trivedi et al., 2021; Bai et al., 2022; Zhang et al., 2019). Specifically, rhizosphere microorganisms promote nutrient uptake by releasing available nutrients into the soil via primary or secondary metabolites. In addition, rhizosphere microorganisms enhance plant disease resistance by inhibiting the growth of pathogens or inducing systemic resistance in plants (Stringlis et al., 2018). However, pronounced differences in rhizosphere microbial community structures have been observed among different genotypes, even within the same crop species (Walters et al., 2018). Stress tolerance is an important factor that influences rhizosphere microbial community structure. For instance, the varying resistance of kiwifruit to bacterial canker disease (Fu et al., 2024), and that of tobacco (Zhao et al., 2023; Xia et al., 2024; Yang et al., 2024), mulberry (Dong et al., 2021), and tomato (Choi et al., 2020) to bacterial wilt are linked to differences in microbial community composition.

Throughout their growth cycle, plants are exposed to various abiotic and biotic stresses, such as insect herbivory and pathogen infections. Plants have developed resistance mechanisms to cope with these challenges (Song et al., 2021; Liu et al., 2023). One strategy involves altering the rhizosphere microbial community in response to stress. Plants tend to recruit specific functional microbial groups to enhance their ability to withstand adverse environmental conditions (Chen et al., 2018). For example, plants may recruit nutrient-cycling microbes under nutrient-deficient conditions and disease-suppressing microbes during pathogen attacks (Xun et al., 2021). For instance, maize selectively enhances the colonization of phosphate-solubilizing microbes in the rhizosphere under low-phosphorus conditions (Shao et al., 2021), and rice recruits rhizosphere microorganisms to improve salt tolerance in saline soils (Lian et al., 2020). Similarly, wheat (Yin et al., 2021), maize (Xun et al., 2023), and cabbage (Ping et al., 2024) selectively enrich antagonistic microorganisms in their rhizospheres in response to pathogen attacks.

The exploration of specific functional microorganisms and the “bottom-up” regulatory mechanisms of the rhizosphere microbiome on plant phenotypic traits is of great significance for leveraging beneficial rhizosphere microbes to develop sustainable agriculture. In the case of late blight caused by *P*. *infestans*, previous research primarily focused on the genetic resistance of tomato cultivars and their direct interaction with the pathogen, few studies have directly linked the composition and function of rhizosphere microbiomes to the suppression of *P. infestans*. Unlike previous studies, which primarily focused on pathogen-host interactions, we not only investigated how differences in rhizosphere microbiomes between tomato cultivars with varying late-blight resistance under natural conditions, but also analyzed how they responded to pathogen inoculation. The aim of this study was to elucidate the mechanisms of soil microbiomes in the rhizospheres of different tomato cultivars with varying late-blight resistance and they how respond to pathogen attacks. Furthermore, we also wanted to identify antagonistic microorganisms for the biocontrol of late blight. Our results will contribute to the more precise and integrated management and bio-controlling of tomato late blight disease.

## Materials and methods

### Field site description and experimental designs

The experiment was conducted from October 2023 to January 2024 in a greenhouse at the Vegetable Teaching and Experimental Base of the Agricultural College, Guangxi University (108°17′25″E, 22°51′02″N). The following tomato cultivars were used in this study: ‘Wild Tomato A3’ and ‘Zixia’. The ‘Zixia’ cultivar is highly susceptible to late blight, whereas ‘Wild Tomato A3’ exhibits notable resistance to the disease.

Initially, the seed surfaces were sterilized, soaked, and allowed to germinate before being sown in seedling trays for 30 days of growth. The seedlings were cultivated in pots (30 cm in height, 25 cm in diameter) containing soil with the following properties: pH 6.33, organic matter content 10.56 g·kg^-1^, total nitrogen 0.89 g·kg^-1^, phosphorus 0.78 g·kg^-1^, and potassium 8.51 g·kg^-1^. Available nitrogen, phosphorus, and potassium levels were 20.27, 1.68, and 102.8 mg·kg^-1^, respectively. After 14 days of growth in the pots, the plants were inoculated with late blight pathogen *Phytophthora infestans*. The inoculum was prepared by culturing *P. infestans* on potato dextrose agar medium, and the sporangia were collected. The inoculation procedure involved spraying 10 mL of a 1 × 10_6_ spores/mL solution evenly on each plant. Controls were sprayed with 10 mL of distilled water instead of the inoculum. The plants were then incubated under conditions conducive to disease development (16°C, 90%–95% relative humidity, and 12-h photoperiod). The following four treatment groups were investigated: late blight-resistant tomato cultivar without *P. infestans* inoculation (A3); tomato cultivar highly susceptible to late blight without *P. infestans* inoculation (ZX), late blight-resistant tomato cultivar with *P. infestans* inoculation (A3P), and tomato cultivar highly susceptible to late blight with *P. infestans* inoculation (ZXP).

### Test methods

#### Sample collection

The rhizosphere soil samples were collected on January 18, 2024. Three tomato plants were randomly selected for each treatment. With the tomato plant in the center, the surrounding soil was loosened using a small sterilized spatula, and the tomato plant was carefully uprooted. Large clods were shaken off and the soil attached to the tomato roots was collected as a rhizosphere soil sample (Yang et al., 2023). The rhizosphere soil samples were placed in pre-labeled sterile self-sealing bags and placed in an icebox with ice packs for transport to the laboratory. In the laboratory, impurities were removed from the rhizosphere soil samples, and the soil was sieved through a 2-mm stainless steel mesh for rhizosphere soil microbial analysis (Ling et al., 2015). Each treatment included three biological replicates for rhizosphere soil microbial analysis.

#### DNA extraction and PCR amplification

The total DNA was extracted from the soil samples using the Fast DNA^®^ Spin Kit for Soil (MP Biomedicals, Thomas Irvine, CA, USA) according to the manufacturer’s instructions. The extracted DNA was examined on 1% agarose gel to verify extraction quality, followed by gel electrophoresis. The concentration and purity of the extracted DNA were determined using a NanoDrop 2000 spectrophotometer (Thermo Fisher Scientific, Waltham, MA, USA) (Bulgarelli et al., 2012). Following extraction, the DNA was stored at −20°C for further processing. PCR amplification was performed on an ABI GeneAmp^®^ 9700 (ABI, Los Angeles, CA, USA) using specific primers and sequence types. Targeting the key regions within microbial genomes, we amplified the V3–V4 hypervariable region of the 16S rRNA gene for bacteria and the ITS1 region for fungi. This was achieved using specific primer pairs: 338F (5′-ACTA3PTACGGGAGGCAGCAG-3′) and 806R (5′-GGACTACHVGGGTWTCTAAT-3′) for bacterial samples (Lundberg et al., 2012), and ITS1F (5’-CTTGGTCATTTAGAGGAAGTAA-3’) and ITS2R (5’-GCTGCGTTCTTCATCGATGC-3’) for fungal samples (Adams et al., 2013). The PCR protocol involved an initial denaturation at 95°C for 3 min, followed by denaturation cycles (16S rRNA gene: 27 cycles; ITS gene: 35 cycles) at 95°C for 30 s, annealing at 55°C for 30 s, and extension at 72°C for 72 s. This was concluded with a final extension at 72°C for 10 min and termination at 4°C. The PCR products were detected and recovered using 2% agarose gel electrophoresis. The recovered products were purified using an AxyPrep DNA Gel Extraction Kit (Axygen Biosciences, Union City, CA, USA). The purified products were again detected using 2% agarose gel electrophoresis, and a Quantus™ Fluorometer (Promega, Madison, WI, USA) was subsequently used to ensure the integrity and purity of the samples.

#### Illumina sequencing

Purified amplicons were pooled in equimolar amounts and were then paired-end sequenced on Illumina MiSeqPE300 (bacteria) and MiSeqPE250 (fungi) platforms (Illumina, San Diego, USA) by Majorbio Bio-Pharm Technology Co. Ltd. (Shanghai, China) according to standard protocols. The raw data for the soil bacterial and fungal sequences were deposited in the NCBI Sequence Read Archive database under the accession numbers PRJNA1154786 and PRJNA1154788, respectively.

#### Data processing

Raw FASTQ files were demultiplexed using an in-house Perl script, quality-filtered using fastp version 0.19.6, and merged using FLASH version 1.2.7. The following criteria were used: (i) the reads were truncated at any site receiving an average quality score of <20 over a 50-bp sliding window, truncated reads shorter than 50 bp were discarded, and reads containing ambiguous characters were also discarded; (ii) only overlapping sequences longer than 10 bp were assembled according to their overlapped sequence, the maximum mismatch ratio in the overlapping region was 0.2, and reads that could not be assembled were discarded; (iii) samples were distinguished according to the barcode and primers, and the sequence direction was adjusted for exact barcode matching and two nucleotide mismatches in primer matching. The optimized sequences were then clustered into operational taxonomic units (OTUs) using UPARSE 7.1 with a 97% sequence similarity level. The most abundant sequence of each OTU was selected as the representative sequence. The taxonomy of each OTU representative sequence was analyzed using RDP Classifier version 2.11 against the 16S rRNA gene database (Release138 http://www.arb-silva.de) and fungi Unite database (Release 8.0 http://unite.ut.ee/index.php) using a confidence threshold of 0.7 (Zhou et al., 2024).

### Statistical analysis

Bioinformatic analysis of the soil microbiota was performed using the Majorbio Cloud platform (https://cloud.majorbio.com). Based on OTU information, alpha diversity indices, including observed OTUs, Shannon index, and Chao richness, were calculated using Mothur v1.30.1. Similarities among the microbial communities in different samples were determined using principal coordinate analysis (PCoA) based on Bray–Curtis dissimilarity using the Vegan v2.5-3 package. Linear discriminant analysis (LDA) effect size (LEfSe) (http://huttenhower.sph.harvard.edu/LEfSe) was used to identify significantly abundant taxa (phylum to genera) of bacteria and fungi among the different groups (LDA score > 3.5, *P* < 0.05). FAPROTAX software (1.2.1) was used to predict the functions of the bacterial communities, whereas FUNGuild software (http://www.funguild.org/) was used to predict the functions of fungal communities.

## Results

**Figure 1:**
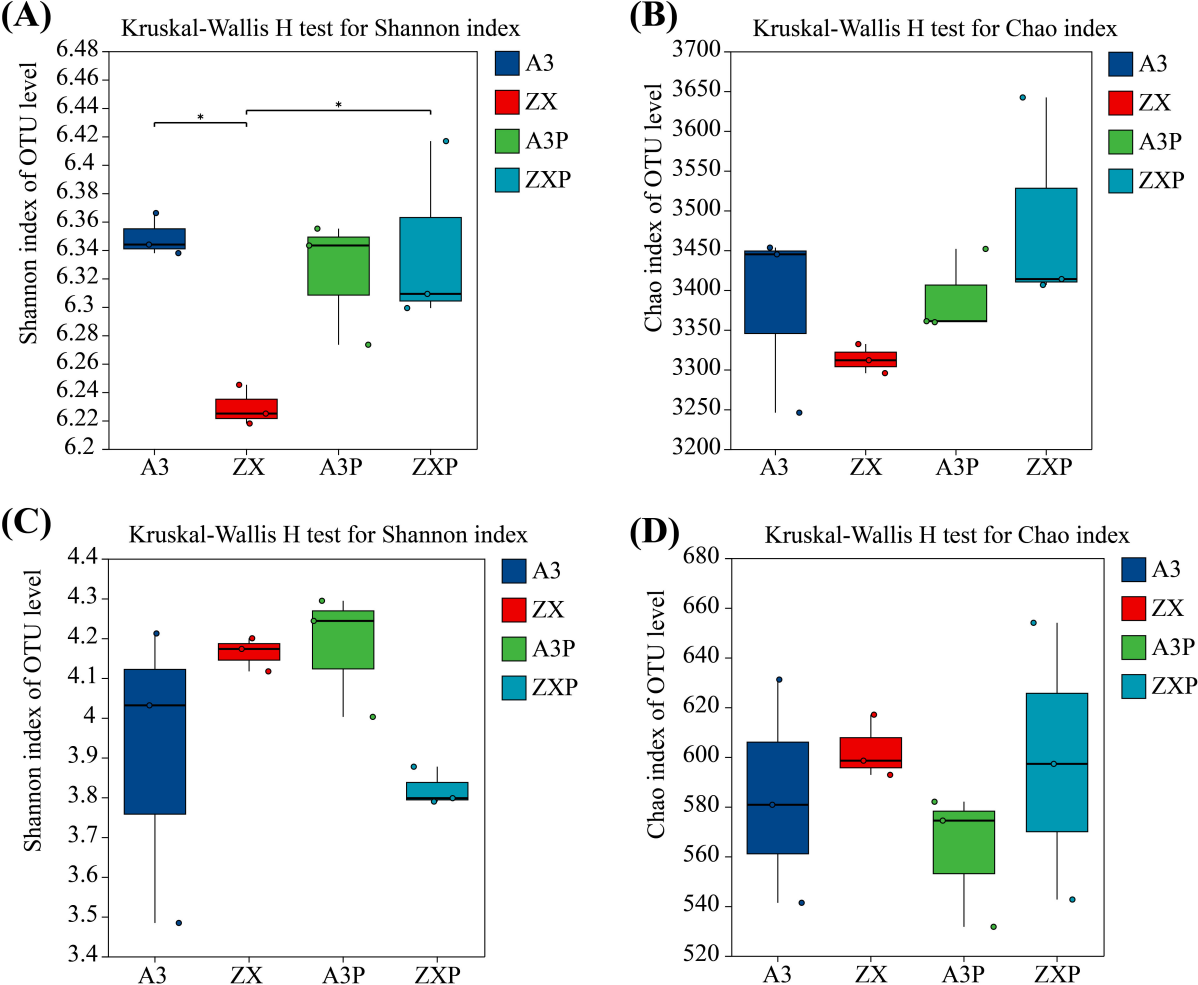
Alpha diversity index of the bacterial **(A, B)** and fungal **(C, D)** communities in the rhizospheres of A3, ZX, A3P, and ZXP plants. A3, late blight-resistant tomato cultivar without *Phytophthora infestans* inoculation; ZX, tomato cultivar highly susceptible to late blight without *P. infestans* inoculation; A3P, late blight-resistant tomato cultivar with *P. infestans* inoculation; ZXP, tomato cultivar highly susceptible to late blight with *P. infestans* inoculation.

As shown in **Figure 1A**, in the absence of *P*. *infestans* inoculation, the Shannon index of the soil bacterial communities in the rhizospheres of A3 was markedly higher than that of ZX. However, the Chao index of soil bacterial richness in the rhizospheres was not significantly different between A3 and ZX (**Figure 1B**). The Shannon and Chao indices of the soil fungal communities in the rhizospheres of A3 were lower than those of ZX; however, no significant differences were observed (**Figures 1C, D**).

Additionally, under *P*. *infestans* inoculation, the Shannon index of the soil bacterial and fungal communities in the rhizospheres of A3P was higher than that of ZXP; however, no significant differences were observed between them (**Figures 1A, C**). In addition, the Chao indices of soil bacterial and fungal richness in the rhizospheres were not significantly different between A3P and ZXP (**Figures 1B, D**).

These results indicated that under natural conditions, the bacterial diversity in the rhizospheres of the resistant cultivar (A3) was markedly higher than that of the susceptible cultivar (ZX). However, bacterial richness and fungal diversity and richness in the rhizospheres were not significantly different from each other. After *P*. *infestans* inoculation, bacterial diversity and richness in the rhizospheres of the resistant cultivar (A3P) decreased, whereas bacterial diversity in the rhizospheres of the susceptible cultivar (ZX) markedly increased. Moreover, after *P*. *infestans* inoculation, soil fungal diversity increased, whereas soil fungal richness decreased in the rhizospheres of the resistant cultivar (A3P). In contrast, soil fungal diversity decreased; however, their richness did not significantly change in the rhizospheres of the susceptible cultivar (ZXP).

**Figure 2:**
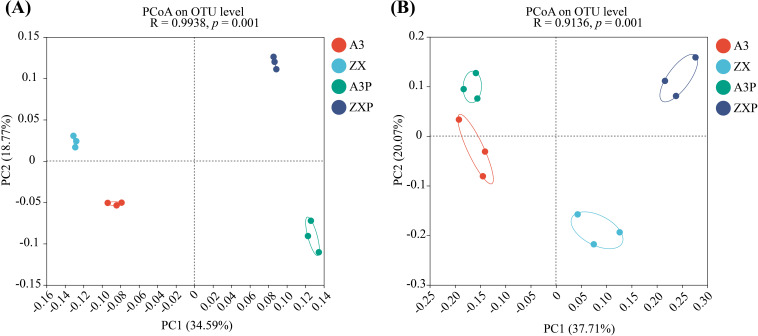
*Beta* diversity indexes of the **(A)** bacterial and **(B)** fungal communities in the rhizospheres of A3, ZX, A3P, and ZXP plants. A3, late blight-resistant tomato cultivar without *Phytophthora infestans* inoculation; ZX, tomato cultivar highly susceptible to late blight without *P. infestans* inoculation; A3P, late blight-resistant tomato cultivar with *P. infestans* inoculation; ZXP, tomato cultivar highly susceptible to late blight with *P. infestans* inoculation; PCoA, principal coordinate analysis; OTU, operational taxonomic unit; PC, principal component.

Based on the Bray–Curtis distance, PCoA was used to visualize the compositional characteristics of the bacterial and fungal communities in the rhizospheres of A3, ZX, A3P, and ZXP plants. Distinct clustering of rhizospheric bacterial and fungal communities was observed among A3, ZX, A3P, and ZXP plants (**Figure 2**). Further analysis should be conducted to explore the specific differences in community composition.

**Figure 3:**
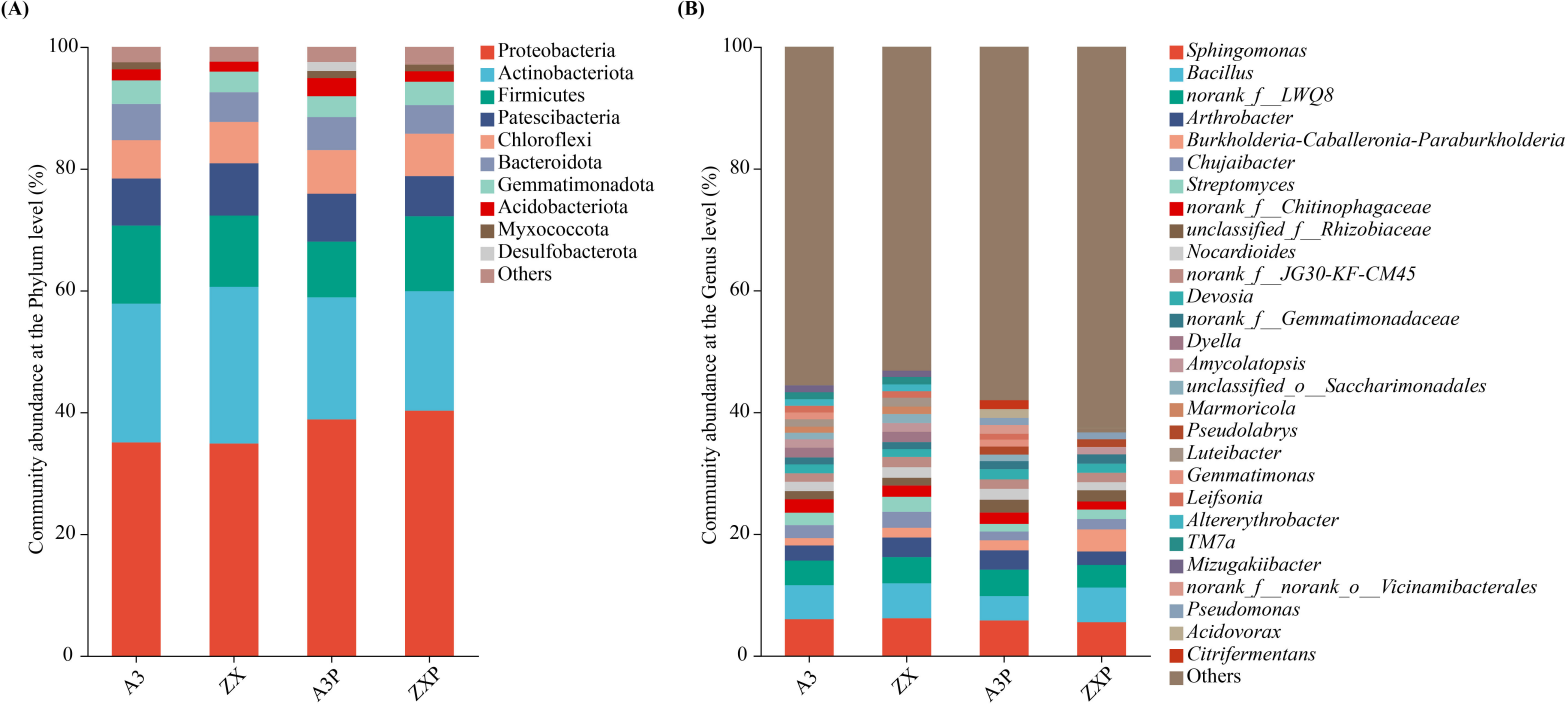
Rhizospheric bacterial compositions at the phylum **(A)** and genus **(B)** levels among A3, ZX, A3P, and ZXP. A3, late blight-resistant tomato cultivar without *Phytophthora infestans* inoculation; ZX, tomato cultivar highly susceptible to late blight without *P. infestans* inoculation; A3P, late blight-resistant tomato cultivar with *P. infestans* inoculation; ZXP, tomato cultivar highly susceptible to late blight with *P. infestans* inoculation.

As shown in **Figure 3A**, at the phylum level, the top five dominant bacterial phyla (relative abundance ≥1%) were consistent, namely Proteobacteria, Actinobacteriota, Firmicutes, Patescibacteria, and Chloroflexi. However, other dominant bacterial phyla, such as Desulfobacterota, were the unique dominant soil bacterial phyla in the rhizospheres of A3P, and Myxococcota was absent in the rhizospheres of ZX.

As shown in **Figure 3B**, the top 10 dominant soil bacterial genera (relative abundance ≥1%) in the rhizospheres of A3 were *Sphingomonas*, *Bacillus*, *norank_f:LWQ8*, *Arthrobacter*, *norank_f:Chitinophagaceae*, *Chujaibacter*, *Streptomyces*, *Dyella*, *Nocardioides*, and *Devosia*. In contrast, *Sphingomonas*, *Bacillus*, *norank_f:LWQ8*, *Arthrobacter*, *Chujaibacter*, *Streptomyces*, *norank_f:Chitinophagaceae*, *Nocardioides*, *norank_f:JG30-KF-CM45*, and *Dyella* were the top 10 dominant soil bacterial genera in the rhizospheres of ZX. Additionally, *Sphingomonas*, *norank_f:LWQ8*, *Bacillus*, *Arthrobacter*, *unclassified_f:Rhizobiaceae*, *norank_f:Chitinophagaceae*, *Nocardioides*, *Devosia*, *Burkholderia-Caballeronia-Paraburkholderia*, and *norank_f:JG30-KF-CM45* were the top 10 dominant soil bacterial genera in the rhizospheres of A3P. In contrast, *Bacillus*, *Sphingomonas*, *norank_f:LWQ8*, *Burkholderia-Caballeronia-Paraburkholderia*, *Arthrobacter*, *unclassified_f:Rhizobiaceae*, *Chujaibacter*, *Streptomyces*, *norank_f:Gemmatimonadaceae*, and *norank_f:JG30-KF-CM45* were the top 10 dominant soil bacterial genera in the rhizospheres of ZXP.

These results suggest that soil bacterial composition in the rhizospheres of tomato cultivars with different resistance abilities to late blight could be altered by *P. infestans* inoculation. In particular, the relative abundances of Firmicutes and Patescibacteria decreased in the rhizospheres of A3P; however, they increased in the rhizospheres of ZXP. Moreover, *Citrifermentans* was uniquely enriched in the rhizospheres of A3P. In contrast, the relative abundance of *Burkholderia-Caballeronia-Paraburkholderia* markedly increased by 125.6% in ZXP compared to that in ZX. Moreover, *Pseudolabrys* and *Pseudomonas* were enriched in the rhizospheres of different tomato cultivars only after *P. infestans* inoculation.

**Figure 4:**
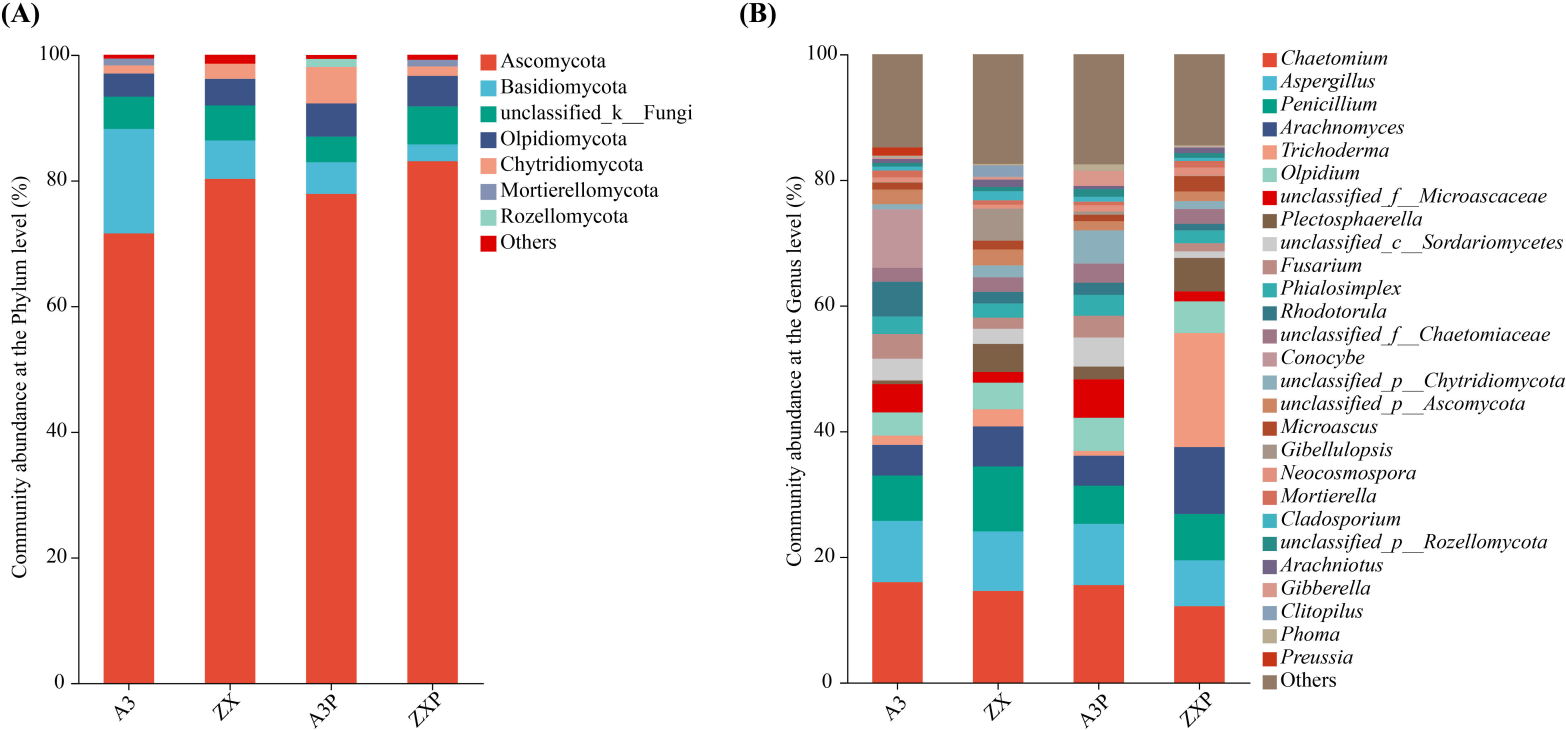
Rhizospheric fungal compositions at the phylum **(A)** and genus **(B)** levels among A3, ZX, A3P, and ZXP. A3, late blight-resistant tomato cultivar without *Phytophthora infestans* inoculation; ZX, tomato cultivar highly susceptible to late blight without *P. infestans* inoculation; A3P, late blight-resistant tomato cultivar with *P. infestans* inoculation; ZXP, tomato cultivar highly susceptible to late blight with *P. infestans* inoculation.

As shown in **Figure 4A**, Ascomycota, Basidiomycota, unclassified_k:Fungi, Olpidiomycota, and Chytridiomycota were the top five common soil dominant fungal phyla (with relative abundance ≥1%) in the rhizospheres of tomato cultivars with varying resistance to *P*. *infestans*.

As shown in **Figure 4B**, the top 10 dominant soil fungal genera (with relative abundance ≥1%) in the rhizospheres of A3 were *Chaetomium*, *Aspergillus*, *Conocybe*, *Penicillium*, *Rhodotorula*, *Arachnomyces*, *unclassified_f:Microascaceae*, *Fusarium*, *Olpidium*, and *unclassified_c:Sordariomycetes*. In contrast, *Chaetomium*, *Penicillium*, *Aspergillus*, *Arachnomyces*, *Gibellulopsis*, *Plectosphaerella*, *Olpidium*, *Trichoderma*, *unclassified_p:Ascomycota*, and *unclassified_c:Sordariomycetes* were the top 10 dominant soil fungal genera in the rhizospheres of ZX. Additionally, *Chaetomium*, *Aspergillus*, *unclassified_f:Microascaceae*, *Penicillium*, *Olpidium*, *unclassified_p:Chytridiomycota*, *Arachnomyces*, *unclassified_c:Sordariomycetes*, *Fusarium*, and *Phialosimplex* were the top 10 dominant soil fungal genera in the rhizospheres of A3P. In contrast, *Trichoderma*, *Chaetomium*, *Arachnomyces*, *Penicillium*, *Aspergillus*, *Plectosphaerella*, *Olpidium*, *Microascus*, *unclassified_f:Chaetomiaceae*, and *Phialosimplex* were the top 10 dominant soil fungal genera in the rhizospheres of ZXP.

These results showed that the relative abundances of Ascomycota and Olpidiomycota increased, and the relative abundance of Basidiomycota decreased in the rhizospheres of A3P and ZXP. Furthermore, the relative abundance of Chytridiomycota markedly increased in the rhizospheres of A3P, whereas it declined in the rhizospheres of ZXP plants. Moreover, Rozellomycota is a unique soil-dominant fungal phylum in the rhizospheres of A3P. At the genus level, *Conocybe* was specifically enriched only in the rhizospheres of A3. However, after inoculation with *P. infestans*, *Conocybe* was absent. *Gibellulopsis* was enriched in the ZX rhizospheres. After *P. infestans* inoculation, the relative abundance of *Trichoderma* in ZXP rhizospheres increased by 607.75% compared to that in ZX rhizospheres.

**Figure 5:**
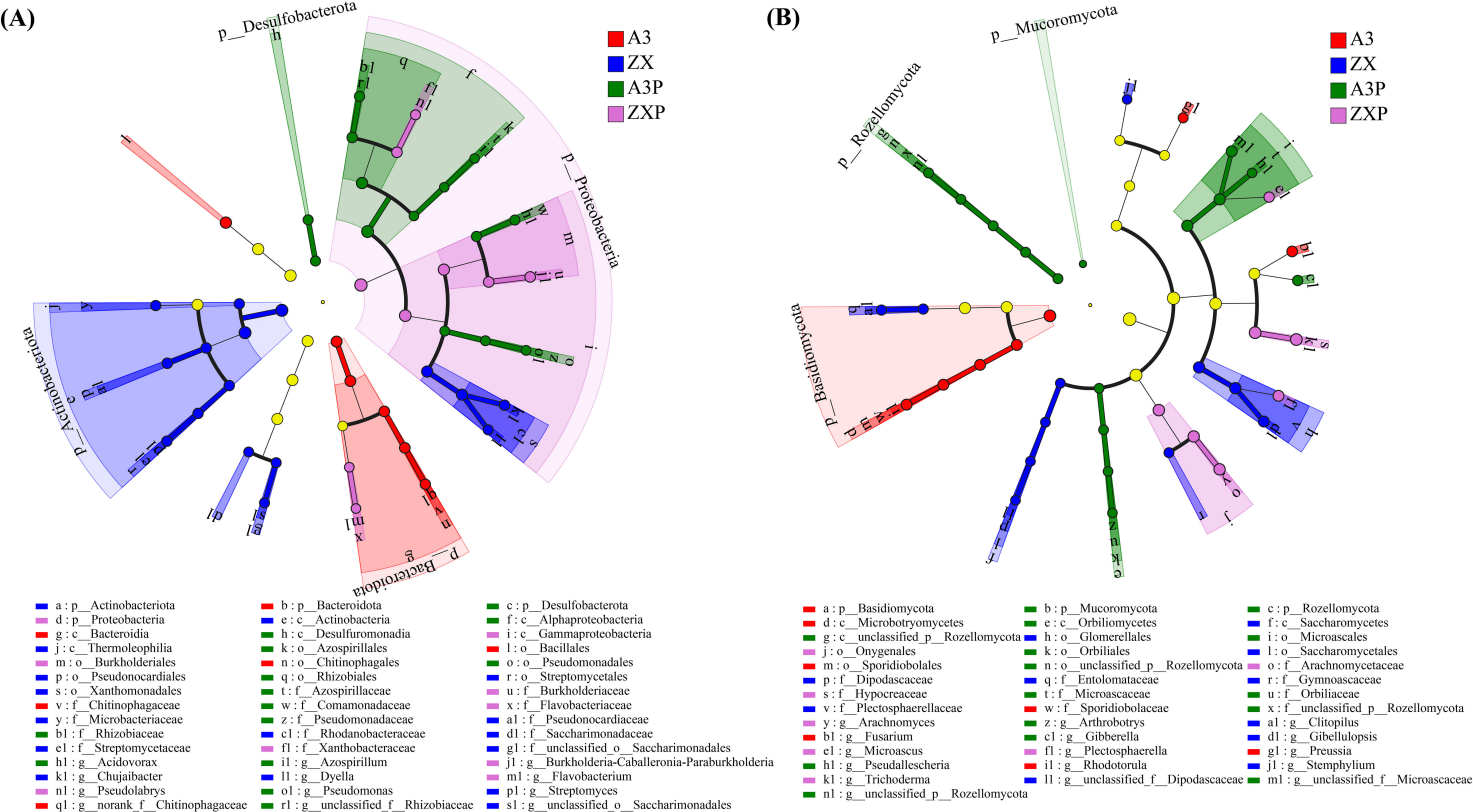
LEfSe analysis of significant abundance of rhizospheric bacteria **(A)** and fungi **(B)** among A3, ZX, A3P, and ZXP plants. A3, late blight-resistant tomato cultivar without *Phytophthora infestans* inoculation; ZX, tomato cultivar highly susceptible to late blight without *P. infestans* inoculation; A3P, late blight-resistant tomato cultivar with *P. infestans* inoculation; ZXP, tomato cultivar highly susceptible to late blight with *P. infestans* inoculation; LEfSe, linear discriminant analysis effect size.

**Figure 6:**
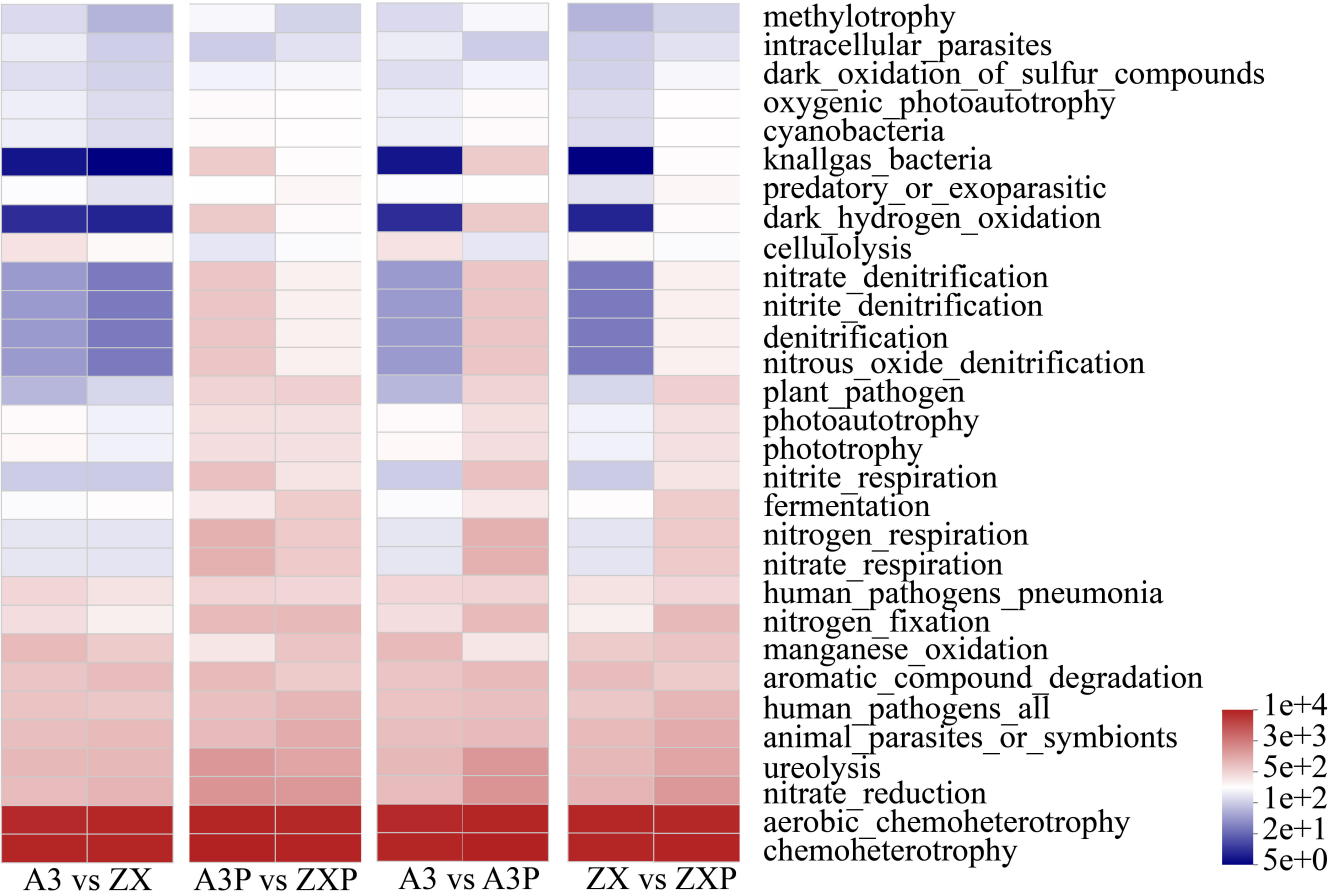
FAPROTAX functional prediction of rhizospheric bacteria in A3, ZX, A3P, and ZXP plants. A3, late blight-resistant tomato cultivar without *Phytophthora infestans* inoculation; ZX, tomato cultivar highly susceptible to late blight without *P. infestans* inoculation; A3P, late blight-resistant tomato cultivar with *P. infestans* inoculation; ZXP, tomato cultivar highly susceptible to late blight with *P. infestans* inoculation.

As shown in **Figure 5A**, 6, 16, 14, and 9 bacterial taxa were identified as biomarkers in the A3, ZX, A3P, and ZXP rhizospheres, respectively. At the phylum level, Bacteroidetes, Actinobacteria, Desulfobacterota, and Proteobacteria were identified as biomarkers in the rhizospheres of A3, ZX, A3P, and ZXP, respectively. At the genus level, *norank_f:Chitinophagaceae* was identified as a biomarker in the A3 rhizospheres, and *Streptomyces*, *Chujaibacter*, and *Dyella* were identified as biomarkers of the ZX rhizospheres. *Acidovorax*, *Pseudomonas*, *unclassified_f:Rhizobiaceae*, and *Azospirillum* were identified as biomarkers in the A3P rhizospheres, whereas *Flavobacterium*, *Pseudolabrys*, and *Burkholderia-Caballeronia-Paraburkholderia* were identified as biomarkers in the rhizospheres of the ZXP plants.

**Figure 7:**
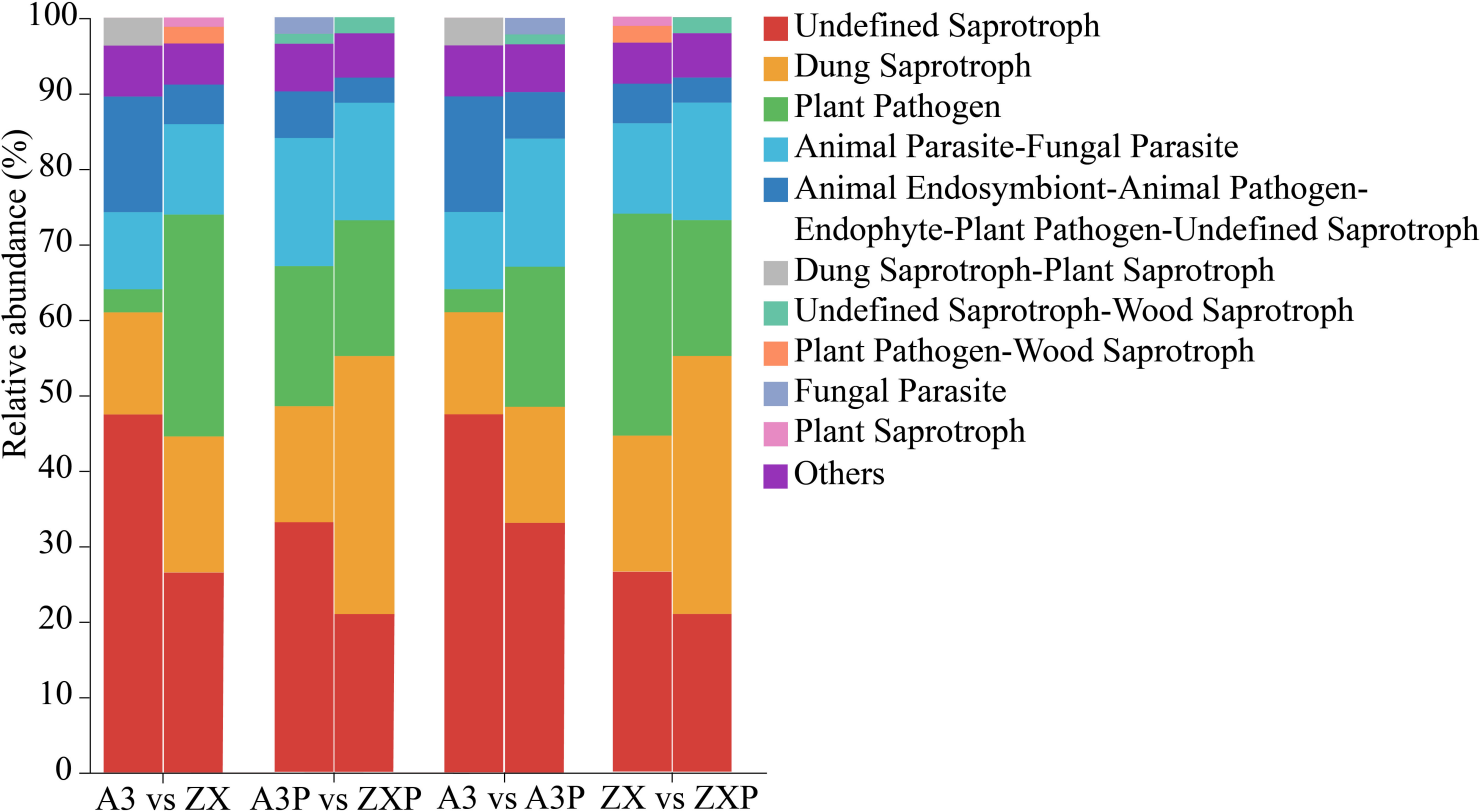
FUNGuild functional predictions of rhizospheric fungi in A3, ZX, A3P, and ZXP plants. A3, late blight-resistant tomato cultivar without *Phytophthora infestans* inoculation; ZX, tomato cultivar highly susceptible to late blight without *P. infestans* inoculation; A3P, late blight-resistant tomato cultivar with *P. infestans* inoculation; ZXP, tomato cultivar highly susceptible to late blight with *P. infestans* inoculation.

As shown in **Figure 5B**, 7, 11, 15, and 7 fungal taxa were identified as biomarkers in the A3, ZX, A3P, and ZXP rhizospheres, respectively. Basidiomycota were markedly enriched in the rhizospheres of A3, whereas Mucoromycota and Rozellomycota were considerably enriched in the rhizospheres of A3P. At the genus level, *Rhodotorula*, *Fusarium*, and *Preussia* were significantly enriched in the rhizospheres of A3, whereas *Gibellulopsis*, *Clitopilus*, and *Stemphylium* were notable enriched in the rhizospheres of ZX. *Gibberella*, *Arthrobotrys*, and *Pseudallescheria* were markedly enriched in the A3P rhizospheres. In contrast, *Trichoderma*, *Arachnomyces*, *Plectosphaerella*, and *Microascus* were considerably enriched in the rhizospheres of the ZXP plants.

As shown in **Figure 6**, the bacterial communities in the A3, ZX, A3P, and ZXP rhizospheres exhibited the highest expression levels during chemoheterotrophy and aerobic_chemoheterotrophy. After inoculation with *P. infestans*, the expression levels of the following 10 functions markedly increased in both A3P and ZXP: knallgas_bacteria, dark_hydrogen_oxidation, nitrate_denitrification, nitrous_oxide_denitrification, nitritedenitrification, denitrification, nitriterespiration, nitrogenrespiration, ureolysis, and nitraterespiration. In addition, the expression levels of A3P were markedly higher than those of ZXP. The expression levels of plant pathogens, nitrogenfixation, and nitrate_reduction also showed notable increasing trends after inoculation, although there were no significant differences between A3P and ZXP. In A3P rhizospheres, the expression levels of cellulolysis and Mn oxidation were considerably lower than those in A3.

FUNGuild predicted the trophic and functional guilds of fungal communities in the A3, ZX, A3P, and ZXP rhizospheres. The rhizospheric fungal communities from all four treatments could be classified into ten guilds. Undefined Saprotroph, Dung Saprotroph, Animal Parasite-Fungal Parasite, Plant Pathogen, Animal Endosymbiont-Animal, Pathogen-Endophyte-Plant, and Pathogen-Undefined Saprotroph were common across all treatments. Among these, the relative abundance of Undefined Saprotrophs decreased considerably after pathogen inoculation, with a more pronounced reduction observed in the resistant varieties. The relative abundance of Plant Pathogens in the rhizospheres of ZX was markedly higher than that in A3, whereas the relative abundance in the rhizospheres of A3P was markedly higher than that in A3. However, the relative abundance in the rhizospheres of ZXP was considerably lower than that in the rhizospheres of ZX. Additionally, Dung Saprotroph-Plant Saprotroph was unique to the rhizospheres of A3, Plant Pathogen-Wood Saprotroph and Plant Saprotroph were specific to the rhizospheres of ZX, and fungal parasites were unique to the rhizospheres of A3P. The Undefined Saprotroph-Wood Saprotroph guild was only found in the rhizospheres of A3P and ZXP (**Figure 7**).

## Discussion

Soil microbes in the rhizosphere are closely associated with plant resistance to stress (Oyserman et al., 2022). Moreover, the inherent natural microbiome of a plant confers resistance at both the individual and community levels (Carrión et al., 2019; Li et al., 2022). According to the well-known “cry-for-help hypothesis,” plants under pathogen attack secrete specific root exudates that promote colonization of beneficial microbes in the rhizosphere (Rolfe et al., 2019). Furthermore, when plants are exposed to adverse conditions, such as drought, salinity, alkalinity, pathogens, or pests, rhizosphere microbes engage in symbiotic interactions that enhance their ability to adapt to these stresses (Philippot et al., 2013; Li et al., 2020). Therefore, understanding the assembly of the rhizosphere microbiome is crucial to elucidate its role in plant stress responses. Therefore, in this study, tomato cultivars with varying resistance to late blight were selected to analyze their natural rhizosphere microbiomes and their responses to pathogen inoculation.

First, we observed that soil bacterial diversity in the rhizospheres of the resistant tomato cultivar was considerably higher than that in the rhizospheres of the susceptible cultivar. In contrast, the soil fungal diversity in the rhizospheres of the susceptible cultivar was higher than that of the resistant cultivar. Similarly, bacterial diversity in Chinese wheat varieties resistant to wheat yellow mosaic virus is higher than that in susceptible varieties (Wu et al., 2021). Additionally, the rhizospheres of *Fusarium* head blight-resistant wheat varieties exhibited markedly higher bacterial diversity than that of susceptible varieties, whereas fungal diversity was lower in the rhizospheres of resistant varieties than in their susceptible counterparts (Li et al., 2023). Moreover, after *P. infestans* inoculation, the rhizosphere bacterial diversity of the susceptible cultivar markedly increased, whereas the changes in the resistant varieties were less pronounced. Soil bacterial diversity was considerably increased in the rhizospheres of diseased plants compared to that in healthy plants (Huang et al., 2019). Simply, *P. infestans* inoculation failed to overcome the defense mechanisms of the resistant tomato cultivar, leaving the plants unaffected by the pathogen. Consequently, the rhizosphere microbiomes of the resistant cultivar remained relatively stable. In contrast, the susceptible tomato cultivar was unable to resist pathogen infection, resulting in pronounced alterations in the rhizosphere microbial diversity. A more diverse and abundant rhizosphere bacterial community may be a key factor contributing to the inherent resistance of tomato cultivars to late blight.

Additionally, dominant soil bacterial genera, such as *Dyella*, *Luteibacter*, *TM7a*, *Mizugakiibacter*, *Altererythrobacter*, and *Marmoricola*, were lost in the rhizospheres of both late blight-resistant and -susceptible cultivars after *P. infestans* inoculation. This indicates that the soil microbial composition in the rhizospheres can be altered by *P. infestans* inoculation, regardless of the cultivar resistance. Some soil bacterial genera, such as *Citrifermentans*, *norank_f:norank_o:Vicinamibacterales*, and *Acidovorax*, were specifically enriched in the rhizospheres of the resistant tomato cultivar. Furthermore, LEfSe analysis identified *Azospirillum*, *Pseudomonas*, *Acidovorax*, and *unclassified_f:Rhizobiaceae* as biomarkers in the rhizospheres of the resistant tomato cultivar. *Azospirillum* synthesizes plant hormones (Iparraguirre et al., 2024) that promote plant growth and development (Lambrecht et al., 2000) and enhance plant stress resistance (Gray and Smith, 2005). Moreover, *Acidovorax* activates plant defense mechanisms and limits pathogen colonization (Emmenegger et al., 2023). Furthermore, *Pseudomonas* is a well-known plant growth-promoting rhizobacterium (PGPR) that inhibits fungal pathogens by releasing lytic enzymes, phenazines, and organic volatiles (De Vrieze et al., 2015; Zhang et al., 2015; Müller et al., 2016). *Pseudomonas* also induces plant defense mechanisms and promotes plant growth under stressful conditions (Kumar and Verma, 2018). In contrast, *Pseudolabrys*, *Flavobacterium*, and *Burkholderia-Caballeronia-Paraburkholderia* were identified as biomarkers in the rhizospheres of the susceptible tomato cultivar after *P. infestans* inoculation. Although *Pseudolabrys* is considered a PGPR that enhances plant growth and health (Peng et al., 2022), *Flavobacterium* also contributes to plant health (Lazcano et al., 2021; Zhou et al., 2022; Chen et al., 2023). The microbe can be isolated from soil and produces antimicrobial substances, such as hydrogen cyanide, chitinase, and siderophores (Belimov et al., 2005; Soltani et al., 2010; Kharade and McBride, 2014). Notably, some *Flavobacterium* species protect tomato plants from pathogenic infections by limiting sugar acquisition through efficient sugar metabolism (Kwak et al., 2018). Furthermore, *Burkholderia-Caballeronia-Paraburkholderia* exhibit plant growth-promoting abilities and antagonistic effects against pathogens (Dendooven et al., 2024). Our results suggest that both the resistant and susceptible tomato cultivars can recruit growth-promoting and biocontrol microorganisms into their rhizospheres upon *P. infestans* inoculation.

Additionally, fungal genera, including *Gibberella*, *unclassified_p_Rozellomycota*, and *Phoma*, were specifically enriched in the rhizospheres of the resistant tomato cultivar after *P. infestans* inoculation. In contrast, *Neocosmospora* was predominantly enriched in the rhizospheres of the susceptible tomato cultivar under the same conditions. *Phoma* protects crops from pathogens by inducing systemic resistance (Sultana et al., 2008), whereas *Neocosmospora* causes stem rot disease in various crops, including peanuts (Sun et al., 2012), mulberries (Zhu et al., 2024), dragon fruit (Zheng et al., 2018), and tomatoes (Kwon et al., 2017). LEfSe analysis further revealed that *Gibberella*, *Arthrobotrys*, and *Pseudallescheria* were identified as biomarkers in the rhizospheres of the resistant tomato cultivar inoculated with *P. infestans*. *Arthrobotrys* can control nematodes and induce plant defense responses against fungal pathogens (Bordallo et al., 2002). *Pseudallescheria* also produce bioactive secondary metabolites that combat various plant pathogens (Ko et al., 2010; Zhu et al., 2020). In contrast, *Trichoderma*, *Arachnomyces*, *Plectosphaerella*, and *Microascus* were identified as biomarkers in the rhizosphere of the susceptible tomato cultivar inoculated with *P. infestans*. *Trichoderma* and its secondary metabolites released into the rhizosphere influence plant growth and nutrition, induce systemic resistance, and contribute to pathogen biocontrol (Hermosa et al., 2012; Kotasthane et al., 2015; Zeilinger et al., 2016). *Trichoderma* has a strong ability to compete with pathogens for nutrients and space in the rhizosphere (Soltani et al., 2010). Additionally, *Trichoderma* produces antibiotics and hydrolytic enzymes, such as chitinase and β-1,3-glucanase, which partially degrade pathogen cell walls and lead to parasitism (Rai et al., 2016). In contrast, *Plectosphaerella* is considered to be responsible for diseases in various plants (Usami et al., 2015; Li et al., 2017; Yang L. et al., 2023).

Under natural conditions, soil bacterial composition and function in the rhizospheres of tomato cultivars with varying levels of resistance were similar, with no significant differences. However, after pathogen inoculation, the functional expression levels of the rhizosphere soil bacterial communities markedly increased for 10 bacterial functions: knallgas_bacteria, dark_hydrogen_oxidation, nitrate_denitrification, nitrous_oxide_denitrification, nitrite_denitrification, denitrification, nitrite_respiration, nitrogen_respiration, ureolysis, and nitrate_respiration. Notably, the expression levels in the rhizospheres of the resistant tomato cultivar were markedly higher than those in the susceptible tomato cultivar. In addition, under natural conditions, the relative abundance of pathotrophic fungi in the rhizospheres of susceptible tomato cultivar was considerably higher than that in the rhizospheres of resistant tomato cultivar. Conversely, after *P. infestans* inoculation, the relative abundance of saprotrophic fungi increased in the rhizospheres of the susceptible cultivar. Our results are consistent with those of previous studies that have reported an increase in the abundance of soil saprotrophic fungi in the rhizospheres of diseased plants (Wang et al., 2024).

These findings suggest that tomato resistance to late blight is closely linked to the functional profiles of the rhizosphere soil microbial communities. Under natural conditions, the low abundance of pathogenic microorganisms, combined with the highly expressed bacterial functions following pathogen inoculation, play a positive role in resisting pathogen invasion.

## Conclusion

Under natural conditions, soil bacterial diversity in the rhizospheres of the late blight-resistant tomato cultivar was considerably higher than that in the susceptible cultivar, whereas fungal diversity was higher in the susceptible cultivar. Additionally, the low abundance of pathogenic microorganisms in the rhizospheres of the resistant cultivar coupled with the high functional expression of bacterial communities under *P. infestans* inoculation played a positive role in resisting pathogen invasion. Furthermore, *Pseudomonas*, *Azospirillum*, *Acidovorax*, *Phoma*, *Arthrobotrys*, *Pseudallescheria*, *Pseudolabrys*, *Flavobacterium*, *Burkholderia-Caballeronia-Paraburkholderia*, and *Trichoderma* were identified as functional microorganisms with potential roles in protection against late blight. In contrast, the enrichment of *Neocosmospora* and *Plectosphaerella* in the rhizospheres of the late blight-susceptible tomato cultivar may contribute to the exacerbation of late blight symptoms.

## Data Availability

The datasets presented in this study can be found in online repositories. The names of the repository/repositories and accession number(s) can be found below: https://www.ncbi.nlm.nih.gov/, PRJNA1206999 https://www.ncbi.nlm.nih.gov/, PRJNA1207005.
